# Relationship Between Head Trauma History and Motor Subtype in Early Parkinson’s Disease

**DOI:** 10.3390/medicina62061058

**Published:** 2026-05-30

**Authors:** Hak-Loh Lee, Seong-Min Choi, Soo Hyun Cho, Byeong C Kim

**Affiliations:** 1 Department of Neurology, Chonnam National University Hospital, Gwangju 58128, Republic of Korea; ss10004ok@naver.com (H.-L.L.); k906141h@hanmail.net (S.H.C.); byeong.kim7@gmail.com (B.C.K.); 2 Department of Neurology, Chonnam National University Medical School, Gwangju 61469, Republic of Korea

**Keywords:** Parkinson’s disease, head trauma, motor subtype, tremor-dominant, akinetic-rigid

## Abstract

*Background and Objectives:* Head trauma (HT) has been suggested as a risk factor for Parkinson’s disease (PD), but its impact on the motor and non-motor manifestations remains unclear. We investigated whether patients with early PD and a history of HT differ from those without HT in terms of their motor and non-motor symptom profiles. *Materials and Methods:* We enrolled patients with early PD (disease duration of ≤5 years, modified Hoehn and Yahr stages [mHY] 1–3). HT history was ascertained by structured questionnaire. Motor and non-motor symptoms were evaluated using standardized clinical rating scales. Motor subtypes—tremor-dominant (TD), akinetic-rigid (AR), and mixed—were determined according to established criteria based on the tremor-to-AR score ratio. Subtype distribution and motor scores were compared between HT and non-HT groups using univariate tests, mHY-adjusted ANCOVA, and multivariable models adjusting for age, sex, disease duration, education, mHY stage, MMSE, and BDI score. *Results:* Of 237 patients, 35 (14.8%) reported HT. The HT group had a higher mHY stage than the non-HT group and showed lower total and rest tremor scores on the Unified Parkinson’s Disease Rating Scale, whereas rigidity scores were similar. Bradykinesia and gait/posture scores tended to be higher in the HT group, but these differences did not persist after adjustment for disease severity. The TD subtype was less frequent in the HT group than in the non-HT group (5.7% vs. 30.2%), whereas the AR subtype was more common (82.9% vs. 61.9%). The categorical subtype redistribution remained significant after multivariable adjustment (adjusted OR for AR vs. TD = 4.61, 95% CI 1.28–16.67). No detectable between-group difference in non-motor symptom burden was observed. *Conclusions:* In this single-center cross-sectional cohort, a self-reported history of HT in early PD was associated with a redistribution of motor phenotype categories toward AR-predominant presentations, with no detectable difference in non-motor symptom burden. Given the retrospective binary exposure assessment, these findings should be interpreted as hypothesis-generating, and prospective studies with structured exposure ascertainment are needed to clarify how HT may shape PD motor phenotype expression.

## 1. Introduction

Parkinson’s disease (PD) is a progressive neurodegenerative disorder defined by characteristic features such as bradykinesia, resting tremor, rigidity, and postural instability [1]. Although these symptoms are common across patients, PD shows marked variability in motor manifestations. The best-known motor subtypes include the tremor-dominant (TD) and akinetic-rigid (AR) forms, which differ in primary clinical features, disease progression, treatment response, and underlying neuropathology [2,3,4,5]. Therefore, identifying factors that influence the motor phenotype is crucial for enhancing prognostic accuracy and guiding personalized treatment approaches [6].

PD etiology is multifactorial, involving intricate interactions between genetic predisposition and environmental factors. Emerging evidence indicates that head trauma (HT) is a potentially modifiable risk factor for PD [1,7]. Several epidemiological and case–control studies have reported that individuals with HT history, especially with accompanying loss of consciousness (LOC), have a higher likelihood of developing PD [8,9]. However, most previous investigations have concentrated on disease risk rather than on how prior HT may affect the clinical manifestations of PD. Chronic traumatic encephalopathy, a representative disorder in which parkinsonian symptoms develop in association with HT, is primarily related to tauopathy and clinically characterized by prominent bradykinesia, rigidity, and postural instability, with mild or absent tremor [10]. All these observations imply that HT may also influence the motor phenotype in patients with PD.

To address this gap, we examined whether motor and non-motor symptom (NMS) differences exist between patients with early-stage PD with and without self-reported HT history.

## 2. Materials and Methods

### 2.1. Study Population

This retrospective study included patients with early PD who first attended the Movement Disorders Clinic at our hospital between January 2020 and December 2023. PD diagnosis was established according to the Movement Disorder Society clinical diagnostic criteria for PD [11]. The inclusion criteria encompassed patients with PD at modified Hoehn and Yahr (mHY) stages 1–3 [12] and a disease duration of ≤5 years. Patients with neurodegenerative disorders other than PD or a clinically significant lesion on brain magnetic resonance imaging were excluded. This study was approved by the institutional review board of our hospital and conducted in accordance with the Declaration of Helsinki and its subsequent amendments.

### 2.2. Clinical Evaluation

Demographic and clinical data comprised age, sex, disease duration, years of formal education, and a self-reported history of HT obtained through a structured questionnaire at the first clinic visit.

HT exposure was ascertained as follows. Patients were first asked the screening question, “Have you ever experienced a head injury in the past?” (yes/no). For each patient answering yes, additional details were collected separately for HT events with and without loss of consciousness (LOC), including the number of events, time since injury (in years), cause of injury, and (for LOC-positive events) duration of LOC. The questionnaire did not impose objective severity criteria such as medical evaluation, hospitalization, or imaging confirmation; therefore, the exposure represents self-reported HT history rather than independently verified traumatic brain injury, and we use the term “HT history” throughout to underscore this distinction.

HT events were classified by severity according to the LOC question: patients reporting at least one LOC-positive event were classified as having major HT, and patients reporting only LOC-negative events were classified as having minor HT. Patients with multiple events were classified by the most severe event. This LOC-based severity classification was used in an additional severity-stratified sensitivity analysis. The duration of LOC was queried but had insufficient completeness for quantitative analysis. The temporal interval between HT and PD motor symptom onset was calculated as years from injury to first clinic visit (per the questionnaire) minus the patient-reported duration of motor symptoms at first visit (in years), and was used in additional timing-stratified sensitivity analyses.

Because supplementary exposure parameters were incomplete and the operational definition of HT relied on patient recall, the principal analyses used a binary indicator of self-reported HT history (present vs. absent), with severity based on the LOC-defined major/minor classification and HT-to-PD timing examined in sensitivity analyses (see Section 2.3).

Clinical assessments were performed at the initial clinic visit, prior to the initiation of any dopaminergic therapy (i.e., in the de novo, drug-naïve state). The assessments included mHY staging, along with the Unified Parkinson’s Disease Rating Scale Part II (UPDRS-II; activities of daily living) and III (UPDRS-III; motor examination) scores [13]. NMSs were evaluated using the Non-Motor Symptoms Scale (NMSS) [14]. Global cognitive function and depressive symptoms were assessed through the Mini-Mental State Examination (MMSE) [15] and Beck Depression Inventory (BDI) [16], respectively.

Motor subtypes were classified as tremor-dominant (TD), mixed, or akinetic-rigid (AR) following the ratio approach of Kang et al. [2], adapted from the framework of Schiess et al. [3]. The mean tremor score was calculated as the sum of the four UPDRS-III rest tremor items (right and left upper and lower limbs) divided by 4. The mean akinetic-rigid score was calculated as the sum of 15 UPDRS-III items (comprising 5 rigidity items for neck and four limbs, 8 limb bradykinesia items for finger taps, hand movements, alternating hand movements, and leg agility, bilaterally, arising from chair, and the body bradykinesia item) divided by 15. The tremor-to-AR ratio was then computed: ratio ≥ 1.0 was classified as TD, 0.80–0.99 as mixed, and ≤0.79 as AR.

### 2.3. Statistical Analysis

Clinical characteristics of patients with and without HT were compared using independent-samples *t*-tests for continuous variables and chi-square or Fisher’s exact tests for categorical variables, as appropriate. The Mann–Whitney U test was used as a non-parametric alternative when distributional assumptions were not met, with no change in substantive conclusions. Where appropriate, analyses were repeated by conducting analysis of covariance (ANCOVA) to adjust for the mHY stage as a measure of disease severity, with adjusted *p*-values reported as “adjusted *p*.” For ANCOVA, homogeneity of variance was assessed using Levene’s test, and the homogeneity-of-regression-slopes assumption was evaluated by including the HT-by-mHY interaction term in the model.

To address potential residual confounding beyond mHY stage alone, multivariable models were additionally fitted. For the categorical motor subtype outcome, multinomial logistic regression was performed with TD as the reference category. For continuous motor scores (total tremor, rest tremor, tremor composite, and AR composite), multiple linear regression was performed. Both model classes were adjusted for the same a priori covariate set selected based on clinical relevance to motor phenotype expression: age, sex, disease duration, years of education, mHY stage, MMSE score, and BDI score. MMSE and BDI were included as available measures of cognitive and depressive symptoms that could influence motor phenotype assessment and may partly capture neuropsychiatric differences related to prior HT. Adjusted odds ratios (aORs) and unstandardized regression coefficients (B) are reported with 95% confidence intervals. Multivariable models used complete-case analysis; patients with missing data on any of the covariates were excluded (4 patients were excluded due to missing MMSE or BDI).

For descriptive proportions of motor subtype distributions, 95% confidence intervals were calculated using the Wilson score method. The Benjamini–Hochberg false discovery rate (FDR) procedure was applied to control for multiple comparisons across the eight UPDRS- and subtype-related continuous-score comparisons, with q-values reported alongside unadjusted *p*-values; the BH-FDR procedure was also applied across the nine NMSS domain comparisons for completeness.

Additional sensitivity analyses were conducted to assess robustness of the principal subtype findings: (i) restricting the cohort to patients with PD motor symptom duration ≥ 6 months to mitigate concerns about subtype instability in the earliest disease stages; (ii) restricting HT exposure to events with self-reported LOC (major HT only); (iii) classifying motor subtype using the alternative Jankovic-style scheme (TD/indeterminate/PIGD); and (iv) restricting HT exposure by the temporal interval between HT and PD motor symptom onset (≥1, ≥5, and ≥10 years), to address potential reverse-causation bias from prodromal motor impairment increasing fall risk.

A two-sided *p*-value of <0.05 was considered statistically significant. All analyses were conducted using SPSS Statistics 27.0 (IBM Corp., Armonk, NY, USA).

## 3. Results

A total of 237 patients with PD were enrolled. The mean age was 67.25 ± 10.04 years, and 142 were females and 95 were males. The mean years of formal education were 8.88 ± 4.97 years. The mean disease duration was 14.10 ± 12.45 months, while the mean mHY stage was 1.85 ± 0.70. The mean UPDRS-III and -II scores were 20.94 ± 8.88 and 6.11 ± 5.21, respectively. The average score on MMSE was 26.47 ± 3.64; BDI, 11.22 ± 8.62; and NMSS total, 39.97 ± 28.70 (Table 1).

Of the 237 patients, 35 (14.8%) reported HT history. No significant between-group differences were found in age, sex, disease duration, or UPDRS-III or -II, MMSE, BDI, or NMSS total scores. The mHY stage was significantly higher in the HT group than in the non-HT group (Table 2).

Among the 35 HT-exposed patients, 17 (48.6%) were classified as having major HT and 18 (51.4%) as having minor HT. The temporal interval between HT and the onset of PD motor symptoms varied widely (median 22.7 years; interquartile range 5.3–42.4 years): 32 patients (91.4%) had experienced HT ≥ 1 year before PD motor symptom onset, 26 (74.3%) ≥ 5 years before, and 21 (60.0%) ≥ 10 years before; only three patients had HT within 1 year of (or after) PD motor symptom onset.

Patients with HT had significantly lower total tremor scores than those without HT (1.71 ± 1.96 vs. 2.55 ± 2.27; *p* = 0.042; adjusted *p* = 0.024), and this difference remained statistically significant after adjustment for the mHY stage. These findings indicate a relative paucity of tremor-related manifestations among patients with prior HT. Analysis of the tremor subtypes further showed that the rest tremor score was significantly lower in the HT group than in the non-HT group (0.94 ± 1.28 vs. 1.67 ± 1.77; *p* = 0.005; adjusted *p* = 0.010), whereas postural tremor showed no significant difference between the two groups. In contrast, bradykinesia (10.34 ± 4.31 vs. 8.74 ± 4.38; *p* = 0.047) and gait/posture scores (3.66 ± 2.50 vs. 2.72 ± 1.96; *p* = 0.040) tended to be higher in the HT group than in the non-HT group, although these differences were no longer statistically significant after adjusting for the mHY stage. In addition, rigidity scores were marginally higher in the HT group than in the non-HT group (5.63 ± 2.56 vs. 4.75 ± 2.82) but did not reach statistical significance (Table 3).

A summary of the motor scores according to the subtype domains revealed that patients with HT had lower composite tremor scores (0.43 ± 0.49 vs. 0.64 ± 0.57; *p* = 0.042) and higher AR composite scores (1.20 ± 0.44 vs. 1.01 ± 0.48; *p* = 0.031) than those without HT. However, those differences were attenuated after adjustment for the mHY stage (adjusted *p* = 0.122; *p* = 0.731) (Table 3).

The overall distribution of motor subtypes also differed significantly by HT status (omnibus test across TD/mixed/AR: *p* = 0.010; adjusted *p* = 0.015). The TD subtype was less prevalent in the HT group than in the non-HT group (5.7% [95% CI 1.6–18.6] vs. 30.2% [95% CI 24.3–36.9]), whereas the AR subtype was more frequent (HT group versus non-HT group: 82.9% [95% CI 67.3–91.9] vs. 61.9% [95% CI 55.0–68.3]) and the mixed subtype was comparable (HT group versus non-HT group: 11.4% vs. 7.9%) (Table 3).

To address potential residual confounding beyond mHY stage alone, multivariable models were fitted with adjustment for age, sex, disease duration, education, mHY stage, MMSE, and BDI score (n = 233 with complete covariate data). The categorical subtype association persisted after full multivariable adjustment: HT history was associated with higher odds of the AR phenotype (vs. TD) with adjusted OR = 4.61 (95% CI 1.28–16.67; *p* = 0.020), and the overall HT effect on subtype distribution was significant (likelihood-ratio χ^2^ = 7.37, df = 2; *p* = 0.025) (Table 4). In contrast, multivariable linear regression for continuous motor scores indicated that the unadjusted differences in total tremor (B = −0.69; 95% CI −1.53, 0.14; *p* = 0.103), rest tremor (B = −0.59; 95% CI −1.24, 0.06; *p* = 0.077), tremor composite (B = −0.17; 95% CI −0.38, 0.04; *p* = 0.103), and akinetic-rigid composite (B = 0.05; 95% CI −0.08, 0.18; *p* = 0.441) attenuated to non-significance after multivariable adjustment (Table 4). Across the multivariable analyses, the categorical subtype redistribution retained statistical significance, whereas the unadjusted differences in continuous motor scores did not persist.

Additional sensitivity analyses supported the robustness of the categorical subtype association. Restricting the cohort to patients with motor symptom duration ≥ 6 months (n = 172) preserved the significant HT–subtype association (Fisher’s exact *p* = 0.013). Re-classifying patients using the alternative Jankovic-style scheme (TD/indeterminate/PIGD) likewise preserved the association (Fisher’s exact *p* = 0.040). Restricting HT exposure to events with documented LOC (major HT only) yielded directionally consistent but underpowered effects (Fisher’s exact *p* = 0.101 for subtype distribution; mean total tremor 1.59 vs. 2.55 in non-HT, *p* = 0.089). To address potential reverse-causation bias, we further restricted HT exposure by the temporal interval between HT and PD motor symptom onset: excluding HT events occurring within 1 year of (or after) PD motor symptom onset preserved the categorical association (Fisher’s exact *p* = 0.017), and the association remained directionally consistent at the more stringent ≥5-year and ≥10-year thresholds, although statistical power decreased with sample attrition (Supplementary Table S1).

Comparisons of the scores in the NMSS domains, including cardiovascular/falls, sleep and fatigue, mood and cognition, perception and hallucinations, attention and memory, gastrointestinal, urinary, sexual function, and miscellaneous, demonstrated no statistically significant differences between the HT and non-HT groups (Table 5). Group-specific NMSS total scores were 39.1 ± 28.5 (HT group) and 44.7 ± 30.0 (non-HT group; *p* = 0.303; adjusted *p* = 0.996). After BH-FDR correction across the nine NMSS domain comparisons, all q-values remained non-significant (q range 0.832–0.996) (Table 5).

## 4. Discussion

In this cohort of 237 patients with PD, self-reported HT history was associated with a redistribution of motor phenotype categories rather than a uniform shift in continuous motor scores. The most consistent and interpretable finding was a cross-sectional redistribution characterized by lower TD prevalence and higher AR prevalence among patients with self-reported HT history. Tremor-related expression was attenuated, whereas the apparent group differences in bradykinesia, gait/posture, and the composite AR score appeared to reflect overall disease severity rather than an HT-specific association independent of disease severity. Non-motor symptom burden was comparable between groups; given the modest size of the HT subgroup, this should be read as no detectable difference rather than as positive evidence against an association.

The shift toward an AR-predominant phenotype observed in patients with self-reported HT history is clinically relevant beyond the simple mirror image of reduced tremor expression. The AR phenotype has been consistently associated with a broader, less favorable disease profile in longitudinal cohorts, including faster motor progression, greater cognitive decline, and earlier emergence of postural instability and dementia [5,17]. Recent biomarker work also supports biological distinctness of the AR subtype across non-motor domains, including olfactory function [18]. A recent group-stratified mediation analysis further reported that the non-tremor (PIGD) subtype shows higher levels of depressive symptoms and a stronger association between depression and cognitive decline, including executive function and delayed visual memory, than the tremor-dominant subtype [19]. Within this broader context, our finding that self-reported HT is associated with an AR-leaning phenotype suggests that self-reported HT history may identify a subgroup of patients with a distinct phenotypic context rather than simply a relative tremor-poor presentation.

Previous relevant research has primarily examined disease susceptibility, specifically the increased PD risk following HT, rather than the differences in clinical presentation [9,20,21]. Building upon this literature, our findings suggest that, among individuals who develop PD, self-reported antecedent HT history may be associated with differences in motor phenotype expression. In our cohort, 14.8% of the patients reported prior HT, which was relatively lower than the 21.5% reported in an Italian case–control study [22]. Such discrepancies may be attributed to variations in study design (e.g., single-center, self-administered recall versus multicenter, interviewer-administered standardized questionnaire), differences in operational definitions (e.g., concussion or LOC criteria), and characteristics of the source population.

Although no imaging, biomarker, or pathology data were collected in the present cohort, several mechanisms proposed in the broader literature could plausibly link prior HT to an AR-leaning phenotype, and we discuss them below as candidate hypotheses for future testing rather than as inferences from our data. First, evidence from traumatic brain injury studies has linked HT to persistent astroglial activation and low-grade neuroinflammation, processes that have been hypothesized to influence dopaminergic vulnerability and basal ganglia–cortical circuitry [23,24]. Furthermore, the sustained elevation of glial fibrillary acidic protein levels following injury may support a chronic astroglial response [25,26]. Second, even mild HT is associated with diffuse axonal injury and microstructural alterations in the white matter (WM) (most consistently in the callosal and frontostriatal tracts), as demonstrated in diffusion tensor imaging (DTI) studies [27,28]. In PD, AR-dominant phenotypes exhibit more extensive WM abnormalities than TD subtypes, particularly in the tracts connecting the basal ganglia, supplementary motor area, and corpus callosum [29]. Graph-theoretical analyses indicate that such alterations reduce structural network efficiency, which may disproportionately impair movement speed and amplitude relative to tremor [30]. Conceptually, the relative preservation of the cerebello-thalamo-cortical tremor pathways, coupled with the disruption of the frontostriatal motor control circuits, could bias motor expression toward non-tremor phenotypes. These hypotheses warrant prospective testing through studies integrating DTI metrics and astroglial biomarkers.

Although NMSs are not included in the traditional motor subtype classifications, they are important determinants of disease prognosis and clinical heterogeneity [6,31]. In our cohort, total and domain-specific NMSS scores did not differ between HT and non-HT groups; after BH-FDR correction across the nine NMSS domain comparisons, all q-values remained non-significant (q range 0.832–0.996). Two interpretations are possible. First, the association between self-reported HT history and phenotype expression may be more readily detectable in motor measures than in NMSS-based non-motor measures in early PD. Second, with only 35 HT-exposed patients, statistical power for domain-specific NMSS comparisons was limited, and the NMSS instrument may have insufficient sensitivity to detect subtle exposure-related effects in early disease. Larger prospective studies with more granular non-motor phenotyping may be needed to determine whether self-reported HT history is associated with subtle non-motor differences that the present cohort was not powered to resolve.

A potential concern is reverse causation: that prodromal motor impairment could increase fall risk and contribute to head injuries subsequently misclassified as antecedent exposures. Several features of our data bear on this possibility. First, the median interval between HT and the onset of PD motor symptoms was 22.7 years (interquartile range 5.3–42.4), with 91.4% of HT events preceding PD motor symptom onset by at least 1 year and 60.0% by at least 10 years; these intervals make it less likely that the majority of reported HT events were caused by motor impairment immediately preceding PD diagnosis. Second, in a sensitivity analysis excluding the three HT events occurring within 1 year of (or after) PD motor symptom onset, the categorical subtype association remained significant (Fisher’s exact *p* = 0.017), indicating that the observed association is unlikely to be explained solely by the small subset of injuries with potentially uncertain timing. At more stringent timing thresholds (≥5 and ≥10 years), effects remained directionally consistent. Although very subtle motor or non-motor changes in the prodromal phase of PD cannot be entirely excluded by self-report, reverse causation is unlikely to fully account for the observed phenotype association, although it cannot be excluded.

Clinically, the observation that patients with self-reported HT history more often present with an AR-predominant phenotype, which has been associated with faster progression and less consistent dopaminergic responsiveness in axial domains in the broader PD literature [32,33], raises hypotheses that warrant prospective testing. Examples include whether early mobility-preserving interventions, individualized dopaminergic optimization, or proactive fall-risk assessment provide differential benefit in this subgroup. The present cross-sectional dataset does not assess intervention response or longitudinal progression and therefore cannot directly support specific clinical recommendations; the implications above should be regarded as hypothesis-generating until tested in dedicated longitudinal or interventional studies. From a research perspective, stratifying participants by HT history could reduce motor phenotype heterogeneity in clinical trials and help clarify treatment effects on axial and rigidity-predominant features.

This study has several limitations. First, HT exposure was self-reported via a structured questionnaire that imposed no objective severity criteria; the resulting binary indicator therefore reflects self-reported HT history rather than independently verified traumatic brain injury, and is subject to recall bias and potential exposure misclassification, the direction of which cannot be fully determined. Although severity (major vs. minor, as defined above) and timing relative to PD motor symptom onset were addressed in additional sensitivity analyses, supplementary parameters such as LOC duration, injury mechanism, and frequency were too incomplete for quantitative analysis, precluding formal dose–response evaluation. Second, despite multivariable adjustment for age, sex, disease duration, education, mHY stage, MMSE, and BDI, residual confounding cannot be excluded; unmeasured factors (such as premorbid lifestyle, alcohol use, occupational exposures, vascular risk burden, and pre-trauma cognitive or motor reserve) could plausibly differ between patients with and without self-reported HT history and could independently shape motor phenotype expression. Third, the subgroup with self-reported HT history was small. Only two patients with self-reported HT history were classified as TD, limiting the precision and stability of subtype-specific estimates; subtype distribution findings should therefore be interpreted cautiously despite consistency across sensitivity analyses. The small subgroup also limits power for non-motor and severity-stratified analyses, and absence of significant differences in NMSS domains should not be read as positive evidence against an association. Fourth, the cross-sectional design precludes causal inference and does not capture the timing of HT relative to prodromal disease milestones with precision, although the timing-stratified sensitivity analyses partially mitigate this concern. Fifth, the single-center setting may limit generalizability, as injury reporting practices, recall of remote HT events, and exposure patterns may differ across populations and healthcare systems. Finally, mechanistic interpretations regarding astroglial activation, white-matter integrity, and frontostriatal circuitry are derived from the external literature; no imaging, biomarker, or pathology data were collected in this cohort, and these mechanisms should therefore be regarded as candidate hypotheses rather than as inferences from our data.

## 5. Conclusions

In this single-center cross-sectional study, a self-reported history of HT in early PD was associated with a redistribution of motor phenotype categories toward AR-predominant presentation and with reduced tremor severity, particularly rest tremor, without detectable differences in the NMS burden. These findings highlight the potential relevance of antecedent HT to motor phenotype expression in PD; however, given the cross-sectional design and self-reported exposure, they should be regarded as hypothesis-generating, and prospective studies combining structured exposure ascertainment with longitudinal clinical and imaging assessment will be needed to clarify how HT may influence motor phenotype expression in PD.

## Figures and Tables

**Table 1:** Demographic and clinical characteristics of the study patients with Parkinson’s disease (n = 237).

Characteristics	Values
Age (years)	67.25 ± 10.04
Sex (female:male)	142:95
Disease duration (months)	14.10 ± 12.45
Formal education (years)	8.88 ± 4.97
mHY stage	1.85 ± 0.70
UPDRS-III score	20.94 ± 8.88
UPDRS-II score	6.11 ± 5.21
MMSE	26.47 ± 3.64
BDI	11.22 ± 8.62
NMSS total	39.97 ± 28.70

Values are expressed as mean ± standard deviation. mHY, modified Hoehn and Yahr; UPDRS, Unified Parkinson’s Disease Rating Scale; MMSE, Mini-Mental State Examination; BDI, Beck Depression Inventory; NMSS, Non-Motor Symptom Assessment Scale for Parkinson’s disease.

**Table 2:** Clinical characteristics of the study patients with Parkinson’s disease, according to a history or no history of head trauma.

	No HT (n = 202)	HT (n = 35)	*p*-Value
Age (years)	66.82 ± 10.12	69.71 ± 9.34	0.116
Sex (female:male)	124:78	18:17	0.270
Disease duration (months)	14.67 ± 12.33	10.77 ± 12.83	0.087
Formal education (years)	9.02 ± 4.82	8.11 ± 5.7	0.320
mHY stage	1.8 ± 0.68	2.1 ± 0.75	**0.020**
UPDRS-III score	20.54 ± 8.96	23.2 ± 8.13	0.103
UPDRS-II score	5.88 ± 5.02	7.49 ± 6.15	0.092
MMSE	26.6 ± 3.61	25.77 ± 3.78	0.216
BDI	11.1 ± 8.48	11.91 ± 9.49	0.614
NMSS total	39.18 ± 28.47	44.68 ± 29.99	0.303

Values are expressed as mean ± standard deviation. Bold values indicate statistical significance at *p* < 0.05. HT, head trauma; mHY, modified Hoehn and Yahr; UPDRS, Unified Parkinson’s Disease Rating Scale; MMSE, Mini-Mental State Examination; BDI, Beck Depression Inventory; NMSS, Non-Motor Symptom Assessment Scale for Parkinson’s disease.

**Table 3:** Comparison of motor subtype classification and individual scores between the study patients with and without head trauma.

	No HT (n = 202)	HT (n = 35)	*p*-Value	mHY-Adjusted *p*	BH-FDR q
Motor subtypes					
TD	61 (30.2%)	2 (5.7%)	0.010 ^a^	0.015 ^b^	-
Mixed	16 (7.9%)	4 (11.4%)			
AR	125 (61.9%)	29 (82.9%)			
Motor scores on subtypes					
Tremor score	0.64 ± 0.57	0.43 ± 0.49	0.042 ^c^	0.122 ^d^	0.063 ^e^
AR score	1.01 ± 0.48	1.20 ± 0.44	0.031 ^c^	0.731 ^d^	0.063 ^e^
Motor scores on UPDRS					
Total tremor	2.55 ± 2.27	1.71 ± 1.96	0.042 ^c^	0.024 ^d^	0.063 ^e^
Rest tremor	1.67 ± 1.77	0.94 ± 1.28	**0.005 ^c^**	**0.010 ^d^**	**0.040 ^e^**
Postural tremor	0.88 ± 1.06	0.77 ± 1.09	0.575 ^c^	0.543 ^d^	0.575 ^e^
Rigidity	4.75 ± 2.82	5.63 ± 2.56	0.085 ^c^	0.689 ^d^	0.097 ^e^
Bradykinesia	8.74 ± 4.38	10.34 ± 4.31	0.047 ^c^	0.499 ^d^	0.063 ^e^
Gait/posture	2.72 ± 1.96	3.66 ± 2.50	0.040 ^c^	0.246 ^d^	0.063 ^e^

Values are expressed as mean ± standard deviation or frequency (%). Bold values indicate statistical significance at *p* < 0.05. HT, head trauma; TD, tremor-dominant; AR, akinetic-rigid; UPDRS, Unified Parkinson’s Disease Rating Scale; mHY, modified Hoehn and Yahr; ANCOVA, analysis of covariance. ^a^ Fisher’s exact test. ^b^ Multinominal logistic regression (adjusted for mHY stage). ^c^ Independent sample *t*-test. ^d^ ANCOVA (adjusted for mHY stage). ^e^ Benjamini–Hochberg false discovery rate (BH-FDR) correction across the eight continuous-score comparisons.

**Table 4:** Multivariable analysis of the association between head trauma history and motor outcomes.

Outcome	Adjusted Estimate (95% CI)	*p*-Value
Multinomial logistic regression (TD = reference)		
Mixed vs. TD	4.44 (0.81–24.39)	0.085
AR vs. TD	4.61 (1.28–16.67)	**0.020**
Multiple linear regression		
Total tremor	−0.69 (−1.53, 0.14)	0.103
Rest tremor	−0.59 (−1.24, 0.06)	0.077
Tremor composite	−0.17 (−0.38, 0.04)	0.103
AR composite	0.05 (−0.08, 0.18)	0.441

Multinomial logistic regression: estimates are adjusted odds ratios (aORs) for HT vs. no HT, with TD as the reference category. Multiple linear regression: estimates are unstandardized regression coefficients (B) for HT (vs. no HT). All models were adjusted for age, sex, disease duration, education, mHY stage, MMSE score, and BDI score. n = 233 (4 patients excluded due to missing covariate data). Bold values indicate statistical significance at *p* < 0.05. CI, confidence interval; HT, head trauma; TD, tremor-dominant; AR, akinetic-rigid; mHY, modified Hoehn and Yahr; MMSE, Mini-Mental State Examination; BDI, Beck Depression Inventory.

**Table 5:** Comparison of the non-motor symptoms between the study patients with and without head trauma.

NMSS Domain	No HT (n = 202)	HT (n = 35)	*p*-Value	BH-FDR q
D1: Falls/cardiovascular	2.11 ± 3.22	2.37 ± 3.59	0.662	0.993
D2: Sleep/fatigue	7.42 ± 7.29	8.51 ± 8.88	0.427	0.965
D3: Mood/cognition	7.27 ± 8.64	6.31 ± 7.15	0.536	0.965
D4: Perception/hallucination	0.14 ± 0.75	0.14 ± 0.49	0.996	0.996
D5: Attention/memory	2.48 ± 3.21	2.37 ± 2.14	0.854	0.996
D6: Gastrointestinal tract	3.96 ± 5.51	5.31 ± 5.96	0.185	0.832
D7: Urinary	11.81 ± 10.79	15.03 ± 13.95	0.201	0.832
D8: Sexual function	0.42 ± 1.76	0.46 ± 1.88	0.911	0.996
D9: Others	3.37 ± 4.44	2.89 ± 3.04	0.535	0.965

Values are expressed as mean ± standard deviation. All *p*-values are from independent samples *t*-tests; Welch’s *t*-test was used when assumption of equal variance was not met. BH-FDR q indicates q-value after Benjamini–Hochberg false discovery rate correction across the 9 NMSS domain comparisons. NMSS, Non-Motor Symptom Assessment Scale for Parkinson’s disease; HT, head trauma; D, domain.

## Data Availability

Anonymized data is available upon request from the corresponding author.

## Supplementary Materials

The following supporting information can be downloaded at: https://www.mdpi.com/article/10.3390/medicina62061058/s1, Table S1: Pre-specified sensitivity analyses for the head trauma–motor subtype association.
