# Supplementary material for: Relationship Between Head Trauma History and Motor Subtype in Early Parkinson’s Disease
Source: Medicina (Kaunas). 2026 May 30;62(6):1058. doi: 10.3390/medicina62061058 (PMC13303936; doi:10.3390/medicina62061058)
Supplement: Supplementary file 1 [file medicina-62-01058-s001.zip › medicina-4263526-supplementary.pdf]

Supplementary Table S1. Pre-specified sensitivity analyses for the head trauma–motor subtype association.

Sensitivity scenario	HT (n)	No HT (n)	χ^2 p-value
**Primary analysis (full cohort)**	**35**	**202**	**0.010**
(i) Disease duration ≥ 6 months	21	151	0.013
(ii) Major HT only (LOC-positive)	17	202	0.101
(iii) Jankovic-style subtype classification	35	202	0.040
(iv) HT timing ≥ 1 year before PD onset	32	202	0.017
HT timing ≥ 5 years before PD onset	26	202	0.053
HT timing ≥ 10 years before PD onset	21	202	0.134

All p-values are from Fisher's exact test of the HT × motor-subtype contingency table (3 categories: tremor-dominant, mixed, akinetic-rigid; or, for sensitivity (iii), tremor-dominant, indeterminate, postural instability/gait difficulty). Bold indicates the primary analysis. HT, head trauma; LOC, loss of consciousness; PD, Parkinson's disease.
